# Model answers: Rational application of murine models in arthritis research

**DOI:** 10.1002/eji.201746938

**Published:** 2017-12-14

**Authors:** Robert A. Benson, Iain B. McInnes, Paul Garside, James M. Brewer

**Affiliations:** ^1^ Institute of Infection, Immunity and Inflammation College of Medical, Veterinary & Life Sciences Sir Graeme Davies Building University of Glasgow Glasgow UK

**Keywords:** Animal model, Autoimmunity, Experimental arthritis, Rheumatology, Therapy

## Abstract

Advances in targeted immune therapeutics have profoundly improved clinical outcomes for patients with inflammatory arthropathies particularly rheumatoid arthritis. The landscape of disease that is observed and the treatment outcomes desired for the future have also progressed. As such there is an increasing move away from traditional models of end‐stage, chronic disease with recognition of the need to consider the earliest phases of pathogenesis as a target for treatment leading to resolution and/or cure. In order to continue the discovery process and enhance our understanding of disease and treatment, we therefore need to continuously revisit the animal models we employ and assess their relevance and utility in the light of contemporary therapeutic goals. In this review, we highlight the areas where we consider new developments in animal models and their application are most required. Thus, we have contextualised the relevant mouse models and their use within the current concepts of human inflammatory arthritis pathogenesis and highlight areas of need.

## Introduction

Rheumatoid arthritis (RA) is a prototypic inflammatory arthritis – it is a chronic, painful and disabling inflammatory disease principally resulting in damage to joints and surrounding connective tissues. The processes underlying initiation and perpetuation of disease involve a complex interplay between genetic and environmental factors. Consequently, current research focuses on how these factors lead to perturbations in innate and adaptive immune responses, changes in tissue stroma activity, and ultimately to joint disease. Our current knowledge can be summarised in a step‐wise pathogenesis map that depicts distinct phases or processes in RA 1, 2, 3. In the first, genetic and environmental factors contribute to an underlying breach of self‐tolerance in the absence of overt clinical manifestation. This early autoreactivity is evidenced by the presence of rheumatoid factor and anti‐citrullinated peptide antibodies (ACPA) several years prior to emerging tissue pathology 4. Changes in the glycosylation of APCA Fc precede early signs of synovitis and articular damage 5, marking transition to the second stage. Although the events driving this transition remain ill defined, neurovascular, microtrauma or infection dependent mechanisms have been proposed as triggers of synovitis. Finally, perpetuation of inflammation occurs that leads to articular damage typical of chronic established RA and to attendant co‐morbidities.

The continuing elucidation of the pathogenesis map relies on data obtained from clinical and animal model studies. Debate over the validity of animal models can arise when contrasting results arise from human studies, questioning the validity of animal models. The publication of data stating mouse models poorly reflect human genomic inflammatory responses 6 and associated media coverage has further fueled this debate. However, reassessment of the same dataset later demonstrated clear correlations between mouse and human responses 7, highlighting the importance of careful model selection and data analysis for interpretation in the context of human pathology. Animal models have been, and continue to be, essential in testing hypotheses and defining biological mediators and cellular processes involved in human disease. In this regard, their continued importance has been highlighted recently 8, 9, 10, 11. Here we contextualise animal models in line with current RA pathogenesis concepts and highlight areas of unmet need.

## Informed model selection

It is clearly unrealistic to expect a single model of arthritis to fully recapitulate human disease. However, individually they allow molecular and spatiotemporal dissection of various pathological processes of RA development that would otherwise be impractical, or unethical in a clinical setting. Employing combinations of models offers the potential to better mirror the complexities of human disease, reflecting different environmental, genetic and temporal contributions to clinical heterogeneity. Which models to combine will ultimately be dependent on the scientific question being asked. By mapping existing models onto different stages of human disease, appropriate systems can be selected to investigate major steps in disease initiation and progression. Additional benefits of this type of mapping are to highlight knowledge gaps in pathogenesis and hence where new models may be required. The generation of a comprehensive RA pathogenesis map will require in‐depth analysis of existing models and will rely heavily on generation of detailed standardised protocols. The importance of developing pathogenesis maps, the refinement and standardisation of current models and ethical considerations have recently been discussed in detail elsewhere 8, 9. Descriptions of the models discussed in this article can be found in Table 1.

**Table 1:** Characteristics of selected animal models

Model	Brief description
**Collagen induced arthritis (CIA)**	Induced by intradermal immunisation with type II collagen (CII) emulsed in CFA. Incidence and chronicity depend on susceptibility of mouse strain and the CII being used (heterologous or homologous). H‐2q confers disease susceptibility. Use of homologous CII induced relapsing/chronic arthritis. Both autoreactive anti‐CII T and B cells are mounted.
**HLA class II transgenic mice**	Several HLA class II transgenic mice have been generated using different risk and protection associated loci – see 15.
**SKG mouse**	Development of inflammatory arthritis associated with a point mutation in ZAP‐70. T cell mediated disease but also relies upon presence of microflora. Mice develop autoreactive T and B cell responses.
**IL‐1RA^−/−^ mouse**	Deficiency of IL‐1 receptor antagonist results in spontaneous destructive arthritis. Disease is IL‐17 and T cell dependent.
**K/BxN mouse**	Generated by crossing a TcR transgenic specific for a bovine pancreatic ribonuclease peptide with NOD mice. Resulting mice display T and B cell responses to glucose‐6‐phosphate isomerase (G6PI).
**F759 mouse**	Homozygous mutation in the gp130 IL‐6 receptor subunit results in inflammatory destructive arthritis. Disease is CD4 T cell dependent, but does not rely on autoreactivity.
**Collagen antibody induced arthritis (CAIA)**	Induced using cocktail of anti‐CII antibodies and LPS. Results in a self‐limiting polyarthritis. Inducible in most strains of mice.
**K/BxN serum transfer mouse model**	Serum from K/BxN mice induces transient arthritis is various mouse strains including C57BL/6 and BALB/c. Is T and B cell independent.
**TNFα transgenic mice**	Several TNFα‐transgenic mouse lines develop chronic progressive polyarthritis. Responses are independent of T and B cells.
**Antigen induced arthritis (AIA) mouse model**	Induced by immunisation with methylated bovine serum albumin (mBSA) in CFA followed by articular challenge with mBSA alone. Inflammation of the challenged joint only. A variation using adoptive transfer of Th1 TcR transgenic T cells specific for ovalbumin (OVA) and challenge with OVA has similar effects but demonstrates evidence of anti‐CII T and B cell responses. In both instances Inflammation is self‐resolving.

In addition to the clear definition of models relevant to specific disease stages, there must be greater appreciation of processes regulating transition from stage to stage. While key cells and molecules contributing to pathology have been highlighted, we lack knowledge pertaining to the underlying dynamics of the immune response. Candidate triggers can be highlighted by correlative patient studies but the development of current and new models will be critical in defining mechanisms by which they drive pathogenesis.

## Breach of self‐tolerance

With successes in the treatment of established RA, one critical therapeutic objective is now restoration of antigen‐specific tolerance in RA 12, 13, 14 and associated drug free remission. A hurdle in developing such revolutionary therapeutics is our lack of mechanistic understanding of breach of self‐tolerance and a paucity of arthritis models allowing detailed analysis of processes that regulate tolerance in vivo.

Critical roles for the adaptive immune system in the development of RA are supported in part by predisposing genetic traits. In addition to the long‐standing association with human leukocyte antigen (HLA)‐DR4 alleles, genome wide scanning studies implicate *ptpn22*, *cd40*, *ctla4* and *cd28* in disease pathogenesis. Yet a comprehensive picture integrating these susceptibility loci with mechanisms leading to breach of self‐tolerance in preclinical RA is lacking.

### HLA class II transgenic mice

HLA class II mouse models are relevant to initial breach of tolerance mechanisms including shaping of the TCR repertoire and profiling T cell epitopes arising from autoantigens 15. Similar MHC class II associated pathogenesis is seen in collagen induced arthritis (CIA) 16 where susceptibility is linked with the H2‐A^q^ haplotype 17.

### PTPN22 polymorphisms

Models of additional risk associated alleles include those relating to polymorphisms in protein tyrosine phosphatase non‐receptor type 22 (*PTPN22*). PTPN22 encodes lymphocyte tyrosine phosphatase (Lyp), with the 1858 C to T polymorphism representing one of the highest risk associations after HLA class II 18, 19. Lyp functions as a critical negative regulator of T cell receptor signal transduction and is thus poised to influence the outcome of T cell recognition of cognate antigen. Several approaches to developing murine models to assess the contribution of this risk allele include Ptpn22 knockout, knock in of Ptpn22‐R619W (analogous to the human polymorphism) and transgenic overexpression of the human risk variant (Lyp620W). Details of these models have recently been reviewed, describing specific immune perturbations and parallels to human disease 19. Despite discrepancies between the models, they do provide a platform to assess altered antigen receptor responsiveness, thymic selection, T cell differentiation and ultimately breach of self‐tolerance driving the contribution of *ptpn22* polymorphisms to RA pathogenesis. Yet these risk allele models do not spontaneously develop overt arthritis unless in the context of an additional autoreactive TcR transgene or injection of arthritogenic antigen in adjuvant. Additional factors are required to work in tandem with genetic predispositions in the development of autoimmune T and B cell responses.

### SKG mice

SKG mice, which carry a point mutation of ZAP70 gene, spontaneously develop chronic autoimmune arthritis stemming from altered TcR signaling and consequent perturbations in thymic selection events. Notably, these arthritis prone mice require environmental triggers for disease onset. Rearing of SKG mice in specific pathogen‐free or germ‐free conditions requires fungal or intestinal microbiota respectively to establish the development of arthritis 20, 21, 22. Similar observations have been made in other models including IL‐1 receptor antagonist deficient (IL‐1RA^−/−^) 23 and K/BxN mice 24, providing platforms for defining mechanisms of interplay between intestinal dysbiosis and genetic susceptibility. Additional evidence of possible microbial contribution to RA development is seen in the clinical links between periodontal disease and arthritis severity. Notably, prior oral infection with *Porphyromonas gingivalis* was seen to accelerate joint inflammation and systemic markers of inflammation in CIA 25 and SKG mice 26. Mechanisms pertaining to *P. gingivalis* exacerbation of RA are unclear. However, it is postulated that *P. gingivalis* infection contributes to autoreactivity through post‐translational modification of proteins and the development of antibodies against citrullinated peptides (ACPA) 25, a predictive factor in development of RA 4.

The lung has been implicated as an important site mediating environmental contributions to pathogenesis. Notably, smokers are at a higher risk of developing RA and once afflicted will progress more aggressively 27. Smoking and urban pollution can also promote post‐translational modifications of self‐proteins resulting in production of ACPA and anti‐carbamylated proteins (anti‐CarP), both associated with more severe joint damage 27. To date only tentative steps have been made in modeling the role airways have in mediating the breach of tolerance and RA 28. While intranasal delivery of cigarette smoke extract (CSE) was shown to augment development of CIA 29, 30, long‐term exposure to cigarette smoke and nicotine delayed CIA onset 31. Reasons for this discrepancy are unclear as yet. Gathering important immunological data from such models is paramount in understanding the aetiological role of cigarette smoking in breach of self‐tolerance. The combination of experimental lung models with experimental arthritis is an important area for development.

Clearly genetic susceptibility alone is insufficient for the development of autoreactivity. Environmental contributions such as mucosal damage, microbiome perturbations and/or chemical/microbial mediated post‐translational protein modifications contribute to RA risk. Animal models represent an important opportunity to combine these aspects in a fuller exploration of preclinical pathogenesis concepts.

## Synovitis and articular damage

It is clear that patients can exhibit the preclinical autoimmune phenotype for many years in an essentially asymptomatic state. Yet the cues that drive the transition to overt articular inflammation and the mechanisms of immune cell accumulation in the joint remain enigmatic. Biomechanical stress, hypoxia and trauma have been proposed as important factors in transition to articular localization. Physical trauma has been reported as being significantly associated with RA onset 32, consistent with this, physical damage to joints can drive arthritis in murine models.

### F759 mice

Direct joint damage and consequent microbleeding facilitate arthritis development in mice expressing a variant of the IL‐6 signaling transducer gp130 33. Although T cell accumulation in the joint is prerequisite for arthritis development in these mice, recognition of cognate antigen is not. This may initially seem at odds with evidence for autoreactivity driving RA. However, our own studies using an adoptive transfer model of early arthropathy reveals antigen driven arthritis also results in similar accumulation T cells unrelated to joint antigen (Benson RA unpublished data). It is therefore tempting to speculate that autoantigen driven joint inflammation can result in innate recruitment of memory T cells and their non‐specific activation as in the instance of cytokine activated T cells (Tck) 34.

### Collagen induced arthritis (CIA) and serum transfer models (CAIA & K/BxN)

Deposition of immune complexes in articular surfaces has a clear role in promoting local inflammation and recruitment of immune cells. Recent studies employing CIA implicate key roles for Th17 helper activity in suppressing sialtransferase activity in B cells 35. The resulting switch in glycosylation of the Fc component of autoantibodies produces immune complexes able to enhance osteoclastogenesis and the beginning of articular erosions 5, 35, 36. The further impacts of immune complexes on various aspects of immune pathology are reproducibly seen in collagen‐antibody–induced arthritis (CAIA) 37 and K/BxN serum transfer 38 models where high autoantibody titres are readily achievable. These models allow dissection of innate pathways that seed articular inflammation and drive tissue degradation. They have revealed the dynamic response to immune complex formation, the activation of complement activation, neutrophil and monocyte recruitment, mast cell degranulation and contributions of TNFα, IL‐6 and IL‐1. As recipient mice do not develop endogenous T and B cell autoimmune responses, only acute self‐resolving disease develops. However, onset is rapid with a high penetrance and is MHC class II haplotype independent, making these models compatible with various genetically modified strains.

## Established/chronic disease

Chronic joint inflammation is one of the hallmarks of clinical disease, with the majority of patients presenting at this stage. A picture of key cellular and soluble mediators is beginning to emerge based on the success of several biologics such as anti‐CD20, CTLA4‐Ig and TNFα. Despite this, there is a paucity of models displaying chronic disease hampering mechanistic study.

### TNF transgenic mice

TNF‐overexpressing mice are consistently reported as developing chronic pathology with several lines contributing to our understanding of TNFα activity in disease 39. While these models continue to be useful tools in identifying molecular mechanisms of disease resulting from pathological TNF production 39, 40, 41 and evaluation of therapeutic strategies 40, they do not provide a platform to study adaptive contributions to chronicity. TNF^ΔARE^ mice back‐crossed to a RAG1^−/−^ background still develop erosive polyarthritis, indicating a key role for this cytokine downstream of T and B cell responses 42.

### Chronic collagen induced arthritis

Although the CIA model, IL‐RA^−/−^ and SKG mice have been shown to be T cell dependent and model chronic disease, data pertaining to adaptive mechanisms beyond disease onset are scarce.

Activation of the adaptive immune response clearly occurs early in disease onset, yet how T and B cells might contribute to ongoing chronic pathology is unclear. The acute self‐resolving nature of antibody‐induced models suggests that T and B cell activity is required for ongoing production of autoantibodies. Yet even intact models such as CIA do not readily develop chronic disease. Induction of chronic relapsing CIA in DBA/1 mice can be achieved by immunisation with autologous type II collagen. Antibody responses to particular antigenic epitopes of type II collagen have also been implicated in development of chronic CIA 43. C57BL/6 mice demonstrate a marked predisposition to chronic joint inflammation in the absence of regulatory NOX2 activity. Thus, greater temporal dissection of these models is required to define the adaptive immune system's contribution to chronicity.

A final aspect of chronic disease warranting deeper investigation is the contribution of the articular stromal compartment. Synovial fibroblasts (interstitial fibroblasts, macrophage‐like and fibroblast‐like synovial cells), mast cells, lymphatic and blood vessels, make up the joint lining and contribute to its normal function 44. These cells undergo profound changes in response to articular inflammation, supporting joint destruction and perpetuation of inflammatory processes 44. Synovial fibroblasts in particular have received considerable attention in orchestrating immune cell accumulation in the synovial environment. Numerous studies demonstrate that synovial fibroblasts support leukocyte recruitment, activation and survival (reviewed in 44) and have proposed roles in the development of tertiary lymphoid structures (TLS) seen in chronic disease 45. The majority of data supporting this has been derived from *in vitro* work using primary human cells, with the recent genomic profiling of mouse synovial fibroblasts from TNF‐Tg mice demonstrating comparison with human data demonstrates significant commonalities 46.

Clinical observations including elevated lymphangiogenic factors in RA synovium, increased numbers of lymphatic vessels, yet reduced lymphatic flow implicate a role for the lymphatic system in RA pathogenesis 47. The ability of collecting lymphatic vessels to contract and relax (pulse) is vital to lymph drainage by allowing the movement of fluid against pressure gradients. Studies in K/BxN mice demonstrate biphasic lymphatic activity with enhanced lymphatic pulse rates and elevated lymph drainage seen in acute arthritis switching to decreased lymphatic clearance during chronicity 48. Current theories to explain this switch centre on the contribution of nitric oxide (NO) to lymphatic pulse. NO production by lymphatic endothelial cells (LECs) regulates smooth muscle cell (SMC) contractions and hence lymphatic pulse. Disruption of endothelial NO gradients by iNOS production by active CD11b+ leukocytes and by LECs in response to TNF results in constant relaxation of SMCs, loss of pulse and failed lymphatic clearance 47, 49. Thus, articular immune responses can condition the local stroma, prompting retention of inflammatory cells and mediators, perpetuation of inflammatory processes and establishment of chronic erosive arthritis. In this regard, the additional therapeutic targeting of stromal cells may prove necessary for the effective resolution of RA.

## Technological developments

Disease models have been extremely useful in identifying key cell populations and molecules contributing to pathogenesis (for extensive review see 11). While exploitation of the rapidly evolving –omics fields offer phenotyping of arthritis models in unprecedented detail, the “how, when and where” of their functional characterisation will require clarification. Disease mediators can have different effects throughout the course of the response, for example, predisposition to K/BxN arthritis depends on IL‐4, yet this cytokine is dispensable during effector stages 50. Thus, defining the temporal impact of a mediator will facilitate its targeting therapeutically.

Advances in bioimaging equipment and techniques are already adding spatial and temporal information to mouse models of arthritis. Notable examples include multiplexed longitudinal non‐invasive imaging of disease onset and progression 51, 52, 53. Imaging in this context reduces variation introduced when different experimental animals per time point are used, thus there are obvious data quality and ethical benefits. Continued improvements in molecular and cellular imaging techniques will also assist in defining cellular origins, fates and functions, enabling a more complete mechanistic understanding of disease processes 54, 55.

## Conclusion

Criticism of animal models lies often in their inability to fully recapitulate all of the complex processes leading to disease. Cases of conflicting data between mouse and human studies continue to fuel debate (6, 7 and discussed in the context of RA in 11). Nevertheless, important biological concepts in arthritis and autoimmune diseases have been conceived or tested in animal models. The limitations and successes mean that animal models are neither irrelevant nor that they completely model arthritis. They represent powerful tools to be used appropriately in conjunction with human data ‐ defining how to apply these tools in a rational and appropriate fashion is a significant scientific goal. This will allow models to be applied within a conceptual framework where a clear prior knowledge of the molecular interactions and cellular processes of interest will enable informed and rational model selection. In addition to testing disease hypotheses, mouse models enable evaluation of therapeutic targets and approaches 56. This is notable in the context of RA 57, 58 success of this is particularly evident in the instance of TNFα targeted therapies, chiefly validating TNFα as a target and subsequent development of a variety of drugs to target this pro‐inflammatory cytokine 59, 60. Additional benefits come from the aligning of pre‐clinical testing with current pathogenesis maps where relevant animal models allow the efficacy of current and future therapeutics to be determined in the context of different disease phases and combinations, establishing rational therapeutic regimes.

## Conflict of interest

The authors declare no financial or commercial conflicts of interest.

**Abbreviations**
ACPA: anti‐citrullinated peptide antibodies
AIA: antigen induced arthritis
CIA: collagen induced arthritis
CIAI: collagen antibody induced arthritis
HLA: human leukocyte antigen
LEC: lymphatic endothelial cell
Lyp: lymphocyte tyrosine phosphatase
PTPN22: protein tyrosine phosphatase non‐receptor type 22
RA: rheumatoid arthritis
SMC: smooth muscle cell
